# High Rates of Return to Sports Activities and Work After Osteotomies Around the Knee: A Systematic Review and Meta-Analysis

**DOI:** 10.1007/s40279-017-0726-y

**Published:** 2017-04-11

**Authors:** Alexander Hoorntje, Suzanne Witjes, P. Paul F. M. Kuijer, Koen L. M. Koenraadt, Rutger C. I. van Geenen, Joost G. Daams, Alan Getgood, Gino M. M. J. Kerkhoffs

**Affiliations:** 1 Department of Orthopaedic Surgery, Amphia Hospital, Foundation FORCE (Foundation for Orthopedic Research Care and Education), Molengracht 21, 4818 CK Breda, The Netherlands; 2 Department of Orthopaedic Surgery, Academic Medical Center, ACES (Academic Centre for Evidence-based Sports medicine), ACHSS (Amsterdam Collaboration for Health and Safety in Sports), Meibergdreef 9, 1105 AZ Amsterdam, The Netherlands; 3 Coronel Institute of Occupational Health, Academic Medical Center, University of Amsterdam, Meibergdreef 9, 1105 AZ Amsterdam, The Netherlands; 4 Medical Library, Academic Medical Center, University of Amsterdam, Meibergdreef 9, 1105 AZ Amsterdam, The Netherlands; 5 Department of Orthopaedic Surgery, Fowler Kennedy Sport Medicine Clinic, Western University, 3M Centre, 1151 Richmond Street, London, ON N6A 3K7 Canada

## Abstract

**Background:**

Knee osteotomies are proven treatment options, especially in younger patients with unicompartmental knee osteoarthritis, for certain cases of chronic knee instability, or as concomitant treatment for meniscal repair or transplantation surgery. Presumably, these patients wish to stay active. Data on whether these patients return to sport (RTS) activities and return to work (RTW) are scarce.

**Objectives:**

Our aim was to systematically review (1) the extent to which patients can RTS and RTW after knee osteotomy and (2) the time to RTS and RTW.

**Methods:**

We systematically searched the MEDLINE and Embase databases. Two authors screened and extracted data, including patient demographics, surgical technique, pre- and postoperative sports and work activities, and confounding factors. Two authors assessed methodological quality. Data on pre- and postoperative participation in sports and work were pooled.

**Results:**

We included 26 studies, involving 1321 patients (69% male). Mean age varied between 27 and 62 years, and mean follow-up was 4.8 years. The overall risk of bias was low in seven studies, moderate in ten studies, and high in nine studies. RTS was reported in 18 studies and mean RTS was 85%. Reported RTS in studies with a low risk of bias was 82%. No studies reported time to RTS. RTW was reported in 14 studies; mean RTW was 85%. Reported RTW in studies with a low risk of bias was 80%. Time to RTW varied from 10 to 22 weeks. Lastly, only 15 studies adjusted for confounders.

**Conclusion:**

Eight out of ten patients returned to sport and work after knee osteotomy. No data were available on time to RTS. A trend toward performing lower-impact sports was observed. Time to RTW varied from 10 to 22 weeks, and almost all patients returned to the same or a higher workload.

**Electronic supplementary material:**

The online version of this article (doi:10.1007/s40279-017-0726-y) contains supplementary material, which is available to authorized users.

## Key Points

**Table:** 

Most patients return to sports activities after knee osteotomy, with a tendency to lower-impact sports, and most patients return to work at the same or an even higher workload.
Systematic comparison of current literature is hampered by heterogeneity in patient populations, operative techniques, and the overall lack of accounting for possible confounding factors such as physical and mental comorbidities, preoperative sports level and work status, patient motivation, and surgeon’s advice.
Future prospective studies are needed to gain better insight into the reasons patients do not return to sport or work. These studies should correct for confounders and use the pre-symptomatic phase as a reference point when assessing return to sport and work.

## Introduction

Osteotomies around the knee, such as high tibial osteotomy (HTO) and distal femoral osteotomy (DFO), are well-accepted procedures for the treatment of early-stage unicompartmental knee osteoarthritis (OA) due to varus- or valgus malalignment [1–3]. With the rise of knee arthroplasty (KA) surgery in the 1970s, use of these procedures declined rapidly [4, 5] as osteotomies were considered more demanding than KA and the outcomes and complications less predictable [4]. However, KAs clearly also have their limitations, especially for younger patients in terms of the low percentage of patients returning to high-impact activities, and the possible higher risk of polyethylene wear if they do [6, 7]. Thus, since patients with knee OA are becoming younger and wish to perform more demanding high activities [8, 9], osteotomies around the knee have gained renewed attention. The current thought is that a knee osteotomy may postpone or even avoid KA and presumably allow patients to return to more demanding activities, since native joint structures are preserved.

In addition to the high demands of present-day patients, several other reasons exist for the renewed attention on and increased use of osteotomies around the knee. Outcomes from HTO and DFO have significantly improved with new operative techniques, improved fixation devices, updated evidence-based guidelines, and careful patient selection [4, 10, 11]. As a result, several studies have demonstrated distinct relief of pain and significant functional improvements after HTO and DFO [2, 4, 12]. Survival rates of 87–99% at 5 years and 66–84% at 10 years have been reported for HTO [13–15] and of 74–90% at 5 years [16, 17] and 64–82% at 10 years [18–20] for DFO. Given these good results, it is reasonable to first consider a knee osteotomy when indication criteria are suitable [4, 21].

Indications for osteotomies have also been extended. In addition to the treatment of unicompartmental OA, osteotomies around the knee are increasingly performed as a concomitant treatment to correct alignment in ligament reconstruction, articular cartilage restoration procedures, and meniscal repair or transplantation surgery [22–26]. In these patients, who are mostly younger and more active, the function of the osteotomy is to (1) reduce strain on the reconstructed ligament graft or the posterolateral corner in cases of varus alignment or (2) unload the involved compartments and thereby reduce stress to the biological repair tissue and potentially prevent or postpone progression of early knee OA. Good results for these combined procedures in terms of functional outcome and survival have also been reported [23, 26].

Thus, osteotomies around the knee are increasingly performed in younger patients and show good results in unicompartmental OA and in reconstructive knee surgery. Johnstone et al. [27] suggested that, if osteotomies are being promoted for younger patients, it is important that they perform well in terms of return to sport (RTS) and return to work (RTW). However, studies that report on RTS and RTW after osteotomies around the knee are sparse, and a clear message is lacking in the literature. Consequently, the actual extent to which patients RTS and RTW is still largely unknown. Therefore, the purpose of the present study was to systematically summarize the available evidence on the extent to which patients RTS and RTW after osteotomies around the knee as well as timing of the return.

## Methods

### Search Strategy

We used the PRISMA (Preferred Reporting Items for Systematic reviews and Meta-Analyses) guidelines for this systematic review [28]. Before commencing the literature search, a research protocol was developed and agreed upon by all authors. This protocol was published online at the PROSPERO International prospective register of systematic reviews (http://www.crd.york.ac.uk/PROSPERO/; registration number CRD42016029929). The clinical librarian (JD) developed the search strategy in close cooperation with the first author (AH). We used the World Health Organization International Clinical Trials Registry Platform (WHO-ICTRP) database to identify relevant search terms and to search for ongoing clinical trials on our subject. We searched the electronic databases MEDLINE via PubMed and Embase via OvidSP for relevant literature and the Cochrane database for systematic reviews. Searches were performed up until 21 September 2016. In all databases, the following four categories of keywords and related synonyms were used to build a sensitive search strategy and to provide a systematic search: osteotomy, sport, work, and recovery of function. Search terms were truncated using an asterisk (*) to find all terms beginning with a specific word. Within each keyword category, the different synonyms were combined using the Boolean command “OR” and categories were linked with the Boolean command “AND”. The exact details of the search strategy can be found in the Electronic Supplementary Material (ESM) Appendix S1. The reference lists of selected studies were screened to identify additional studies for inclusion. We also performed a forward search using Web of Science to see which of these studies had been referred to by other authors after publication.

### Eligibility Criteria and Study Selection

We used the Rayyan screening tool for systematic reviews to screen titles and abstracts [29]; all abstracts were screened by two independent reviewers (AH, PK). Discrepancies were resolved by discussion; where there was doubt, the article was included in the full-text screening process. One author (AH) then selected suitable studies based on the eligibility criteria established in the research protocol. This selection was then reviewed by a second author (SW), and discrepancies were resolved by discussion. Inclusion criteria were as follows: observational or intervention studies describing patients with malalignment who underwent any type of corrective knee osteotomy for any indication and who were participating in sport activities and/or working before the surgery and intended to RTS and/or RTW after surgery. No language restrictions were used. The primary outcomes were the percentage and number of patients to RTS and RTW, preferably described in terms of level, duration, and frequency. Secondary outcomes included activity-specific outcome measures, namely the Tegner activity score (0–10; higher is better), the Lysholm score (0–100; higher is better), the International Knee Documentation Committee (IKDC) objective score (0–100; higher is better), the University of California, Los Angeles (UCLA) activity score (0–10; higher is better), and the Naal activity score, which investigates pre- and postoperative engagement in 20 different sports activities. The Reichsausschuss für Arbeitszeitermittlung (REFA; German workload classification) Association classification system (from “0 = work with no physical strain” to “4 = work with most heavy physical strain”) was also collected as a work-related outcome measure.

### Methodological Quality

We assessed the risk of bias of the included studies using the Quality in Prognosis Studies (QUIPS) tool [30]. This quality-assessment tool includes six domains of potential bias: (1) study participation, (2) study attrition, (3) prognostic factor measurement, (4) outcome measurement, (5) study confounding, and (6) statistical analysis and reporting. Each domain contains two or more sub-domains that should be rated as “yes,” “partial,” “no,” or “unsure.” The answers to each sub-domain are then combined, leading to a “low,” “moderate,” or “high” risk of bias. The first author (AH) assessed the quality of all included studies; this was then repeated independently by two other authors (PK, KK), who each assessed the risk of bias for half of the included studies. Disagreements were resolved by discussion and, if necessary, involving a third reviewer. The details of the quality assessment can be found in the ESM Appendix S2. We considered a study to have an overall low risk of bias when the methodological risk of bias was rated as low or moderate in all six domains, with at least four domains rated as low. A study was rated as having an overall high risk of bias if two or more of the domains were scored as high. In-between quality was scored as moderate. Results of the studies with a low risk of bias are discussed in the text and those of the studies with a moderate or high risk of bias are presented in the data extraction table (Table 1).

**Table 1:** Return to sports and work after knee osteotomy: data extracted from studies included in the review (*n* = 26)^a^

Study details, design, population [language]	Operation type (+fixation implant)	Rehabilitation protocol	Outcome measures	Preoperative activity + definition	Postoperative activity	RTS + time to RTS	RTW + time to RTW	Confounding factors
Study: Ampollini et al. [42], 1998, Italy [Italian] Design: ret cs, FU NS Population: Pts with chronic anterior laxity and varus malalignment (*n* = 7); age range 24–35; sex 7 M (100%); BMI NS; Co NS	LCW HTO + ACL reconstruction Fixation: plate 5; staples 2	Knee brace for 60 days. CPM from postoperative d3	Sports participation, *n*(%)	0 Definition of pre-op: <1 year before surgery	7	>100%; time to RTS unknown	Unknown	Mentioned, not adjusted for: surgeon’s advice (RTS is not the goal); pre-injury sports level
Study: Bode et al. [25], 2015, Germany Design: ret cohort study, FU: 5.0 ± 0.2 years Population: Pts with cartilage defect medial femoral condyle and varus malalignment >2º (*n* = 40); age 37.6 ± 7.5; sex NS; BMI 25.4 ± 3.4; Co NS	MOW HTO + ACI Fixation: TomoFix	CPM for 6 weeks, up to 4 h/day. Mobilization on postoperative d1. Limited weight bearing for 6 weeks	Lysholm	54.4 ± 18.9	76.2 ± 19.8 (*p* < 0.01)	Unknown	% RTW: unknown Time to RTW: 94.5 ± 77.0 days REFA 1: 68.1 ± 61.4 days REFA 4: 155.0 ± 111.0 days(*p* = 0.023)	Adjusted for in analysis: BMI (>35 not included); workload. Mentioned, not adjusted for: age
			Workload: REFA work (physical strain, *n*(%))					
			0 (without)	–	11			
			1 (small)	–	9			
			2 (moderate)	–	5			
			3–4 (hard, most heavy)	–	14			
				Definition of pre-op: unknown				
Study: Bonnin et al. [43], 2013, France Design: ret, mc (four centers) cohort study; FU 4.2 ± 0.9 years Population: Pts with medial compartment OA and varus malalignment (*n* = 139); age 59.1 (range 24–80); sex 98 M (71%), 41 F (29%); BMI 27.2 ± 4.1; Co: medical limitation (respiratory, cardiac or neurologic): 6	LCW HTO (*n* = 88) MOW HTO (*n* = 51) Fixation: plate 114, blade plate + screws 18, staples 7	NS	Sports participation, *n*(%)			29 (20.8%) more active, 62 (44.6%) same activity level, 45 (33%) less active than before surgery. Time to RTS unknown	Unknown	Adjusted for in analysis: age; motivation. Mentioned, not adjusted for: reasons for no RTS
			Stationary cycling	–	38			
			Road cycling	–	58			
			Stretching	–	54			
			Swimming	–	31			
			Golfing	–	7			
			Sailing	–	6			
			Strength exercise	–	22			
			Dancing	–	10			
			Gymnastics	–	29			
			Hiking	–	60			
			Gardening	–	71			
			C-C skiing	–	14			
			DH skiing	–	35			
			Tennis	–	2			
			Running >500 m	–	6			
			Mean Weiss activity score		5.3 ± 1.2			
			Light	–	5.9			
			Intermediate	–	5.5			
			Strenuous	–	5.1			
				Definition of pre-op: before surgery				
Study: Boss et al. [44], 1995, Switzerland Design: ret cohort study; FU 6.3 years (range 2.6–13.8) Population: Pts with ACL deficiency, existing cartilaginous lesions medial compartment ± medial meniscus lesion (*n* = 20), and varus malalignment (*n* = 27); age 36 (range 19–55); sex 22 M (82%), 5 F (18%); BMI NS; Co NS	ACL reconstruction (BPTB ± LAD, *n* = 13) + HTO (24 LCW, 3 MOW) Fixation: staples, AO-T-plates: semi-tubular plate with long screw in ventral tibial cortex	Dorsal cast and removable circular splint. Immediate passive ROM, early mobilization. Full weight bearing. At 3 months cycling and jogging allowed, at 6–9 months more demanding sports	Activity level	Unknown. Definition of pre-op: pre-trauma and pre-surgery	55% higher at FU than preoperatively 15% lower at FU than preoperatively	94%; 85% returned to same or higher level. Time to RTS unknown	89% had returned to same profession at FU. Time to RTW unknown	Mentioned, not adjusted for: concomitant surgery
Study: Boussaton et al. [41], 2007, France [French] Design: ret cs; FU NS (range 1–10 years) Population: Professional rugby players requiring HTO (*n* = 6); age NS; sex 6 M (100%); BMI NS; Co NS	Valgising HTO (*n* = 4) and varising HTO (*n* = 2) Fixation NS	NS	IKDC	Unknown. Definition of pre-op: pretraumatic	94 (range 86–99)	100% (6/6). Time to RTS unknown	Unknown	NS
Study: Cotic et al. [45], 2015, Germany Design: pro cohort study; FU: 2 years Population: Pts with medial compartment OA and varus malalignment or medial compartment overload combined with localized chondral defects requiring cartilage repair (*n* = 28); age 45 (±11); sex 19 M (70%), 9 F (30%); BMI 25 ± 3; Co NS	(Biplanar) MOW HTO Fixation: second-generation peek-carbon composite plate Concomitant procedures: medial meniscectomy 5, microfracturing 1, OATS 6, ACL reconstruction 1	Active and passive FROM as tolerated directly or after 6 weeks (in microfracture and OATS pts). 20-kg partial weight bearing until 6 weeks, then full weight bearing was allowed	Lysholm (*n* = 27)	51 (40–62)	83 (73–94) (*p* < 0.001)	>100%	Unknown	Mentioned, not adjusted for: fixation type; timing of implant removal
			Tegner (*n* = 27)	5 (3–6)	4 (3–5) (n.s.)	Time to RTS unknown		
			Sports participation, *n*(%)			RTS (%)		
			Overall	24	27	>100		
			Windsurfing	1	1	100		
			Sailing	1	2	>100		
			Dancing	4	4	100		
			Martial arts	1	1	100		
			Basketball	2	1	50		
			Soccer	2	0	0		
			Bowling	1	1	100		
			Badminton	3	0	0		
			Table tennis	2	2	100		
			Tennis singles	3	0	0		
			Golf	1	2	>100		
			Hunting	2	1	50		
			Ice skating	1	1	100		
			Snowboarding	1	0	0		
			C-C skiing	7	6	86		
			Downhill skiing	5	8	>100		
			Aqua fit	0	1	>100		
			Gymnastics	4	5	>100		
			Aerobics	1	0	0		
			Fitness training	9	15	>100		
			Swimming	13	14	>100		
			Mountain biking	6	7	>100		
			Cycling	19	24	>100		
			Climbing	3	3	100		
			Hiking	10	12	>100		
			Inline skating	2	3	>100		
			Jogging	6	7	>100		
			Nordic walking	5	9	>100		
				Definition of pre-op: regular participation in year before surgery				
Study: Dahl et al. [40], 2015, Sweden Design: pro cohort study; FU 10 years Population: Pts with unicompartmental knee OA treated with hemicallotasis HTO technique (medial OA 40; lateral OA 5) (*n* = 45); age 55 (range 35–64); sex 31 M (69%), 14 F (31%); BMI 29 ± 4.5; Co NS	HTO by hemicallotasis technique Fixation: external fixator	Free mobilization allowed. Full weight bearing. PT prescribed individually and related to needs of pt	Level of physical activity, *n*(%) (lifetime/pre-op):			63% RTS (%)	At 2 years: 84%. At 10 years: 49%	Mentioned, not adjusted for: BMI; expectations; pts converted to TKA were excluded from FU; retirement
			6: competitive sports	19/0	0	0		
			5: recreational sports	9/1	3	33		
			4: golf, dancing, hiking, water aerobics	10/9	21	>100		
			3: heavy yard/household work	7/33	6	86		
			2: light yard/household work	0/2	13	>100		
			1: minimal household work, sewing, card games	0/0	1	–		
			0: no household work, TV/reading only	0/0	1	–		
			Working pts, *n*(%)			Time to RTS: unknown		
			Working	43	21			
			Retired	2	23			
			Unemployed	0	0			
			Sick leave	0	1			
				Definition of pre-op: lifetime and pre-op				
Study: De Carvalho et al. [38], 2012, Brazil Design: Cross-sectional cohort study; FU 4 years (range 1.7–9.5) Population: Pts with lateral compartment OA and valgus malalignment (*n* = 26); age 48.6 (range 21–65); sex 8 M (31%), 18 F (69%); BMI NS (<35 kg/m^2^); Co NS	LCW DFO Fixation: dynamic condylar screw (Synthes)	FROM as tolerated without weight bearing. Partial weight bearing after 6 weeks and full 8–12 weeks. RTS after healing of osteotomy and recovery of muscle strength	Sports participation, *n*(%)			89% RTS (%)	88.5% resumed normal work duties at pre-op functional level. Time to RTW: unknown	Mentioned, not adjusted for: age; limited FU; pre-op sports level; surgical technique
			Routine physical activity	15	14	93		
			Soccer	3	3	100		
			Volleyball	1	0	0		
			Tegner	3 (2–7)	3 (1–7) (n.s.)			
			Lysholm	53.1 ± 16.2 (24–95) Definition of pre-op: unknown	77.3 ± 16.7 (29–100) (*p* < 0.001)	Time to RTS unknown		
Study: Dejour et al. [46], 1994, France Design: ret cohort study; FU 3.6 years (range 1–11) Population: Pts with symptomatic chronic ACL deficiency + acquired varus malalignment (*n* = 44); age 29 (range 18–42); sex 27 M (63%), 16 F (37%); BMI NS; Co NS	ACL reconstruction (BPTB ± LET *n* = 34) + HTO (LCW *n* = 37; MOW *n* = 7) Fixation: two staples	Immediate ROM as tolerated. Non-weight bearing for 8 weeks	Sporting level (pre-injury/pre-surgery, *n*(%))			66% RTS (%)	Unknown	Adjusted for in analysis: no differences in RTS between pts with poor outcome and pts with good outcome
			Pivotal contact (e.g. soccer)	30/17	7	23/41		
			Pivotal non-contact (e.g. tennis)	8/4	10	>100		
			Non-pivotal non-contact (e.g. cycling)	3/4	10	>100		
				Definition of pre-op: both pre-injury and pre-surgery		Time to RTS unknown		
Study: Fasching-bauer et al. [47], 2015, Germany Design: Cross-sectional cohort study; FU 1.8 years ± 0.8 Population: Pts with medial compartment OA and varus malalignment (*n* = 43); age 42 ± 11.2; sex 32 M (74%), 11 F (26%); BMI 26.9 ± 3.6; Co NS; concomitant procedures: 13 (OATS 6; partial meniscectomy 4; microfracturing 3)	MOW HTO Fixation: TomoFix	20-kg partial weight-bearing for 2 weeks, swiftly increased from week 2 until full weight bearing. Daily PT was recommended	General sports participation (at least 1 sport)	39/43 (90.7%)	36/43 (83.7%)	92% (no inactive pts started new activities post-op)	94% returned to pre-op workload. Time to RTW: 16.7 ± 15.6 weeks. Group I (high work intensity, *n* = 13): 19.1 ± 9.1 weeks. Group II (moderate, *n* = 12): 20 ± 17.8 weeks. Group III (low, *n* = 15): 11.8 ± 7.8 weeks (*p* = 0.325). No pre- and postoperative changes among groups	Adjusted for in analysis: analgesic use; completion of rehabilitation, cessation of partial weight bearing, workload. Mentioned, not adjusted for: avoidance of potentially harmful activities, limited FU, surgeon’s advice
			Sports activities *n*(%):			RTS (%)		
			Cycling	33	25	76%		
			Hiking	19	16	84%		
			Swimming	18	17	94%		
			Fitness	8	10	> 100%		
			Downhill skiing	10	5	50%		
			Nordic walking	8	6	75%		
			Jogging	8	4	50%		
			Soccer	8	2	25%		
			Gymnastics	5	4	80%		
			Inline skating	6	2	33%		
			Tegner	3.78 ± 1.9	3.7 ± 1.4 (n.s.)			
			Lysholm	Unknown	68.7 ± 23.9	Time to RTS unknown		
				Definition of pre-op: pre-symptomatic				
Study: Gomoll et al. [36], 2009, USA Design: ret study; FU 2 years (range 1–4.2) Population: Pts with ipsilateral chondral defects and meniscal deficiency (*n* = 7); age 32 (range 18–43); sex 5 M (71%), 2 F (29%); BMI NS; Co NS	Meniscus allograft transplantation + cartilage repair + osteotomy: HTO 5, DFO 2 Fixation: NS	Hinged knee brace with CPM for 6 h/day for 6 weeks. Non-weight bearing 6 weeks. ADL activities after 3 months, return to non-contact sports after 4–5 months. No restrictions after 12 months	Lysholm (mean)	34	77 (*p* < 0.01)	100%, 6 to full activities without restrictions, 1 with mild symptoms while playing basketball. Time to RTS unknown	Unknown	Mentioned, not adjusted for: expectation management by surgeon
			IKDC	26 Definition of pre-op: unknown (presumably pre-injury)	63 (*p* < 0.01)			
Study: Hoell et al. [34], 2005, Germany Design: ret cohort study; FU 1.9 years (range 0.7–2.8) Population: Pts with medial compartment OA and varus malalignment treated with MOW HTO [(*n* = 40); age 46.4 ± 8; sex 25 M (63%), 15 F (37%); BMI 30 ± 5.2] or LCW HTO [(*n* = 51); age 52.1 ± 8.4; sex 36 M (70%), 15 F (30%); BMI 29 ± 4.2]; Co: no pts with rheumatic disease	MOW HTO *n* = 40; LCW HTO *n* = 51 Fixation: MOW: Puddu plate; LCW: staples	Limited ROM (0–0–90°) first 6 weeks	Lysholm (range)			Unknown. Time to RTS unknown	RTW unknown. Time to RTW: MOW: 13.9 weeks; LCW: 13.6 weeks (*p* = n.s.)	Adjusted for in analysis: type of osteotomy. Mentioned, not adjusted for: fixation type (Puddu plate, with pain at implant site); rehabilitation
			MOW	46 (25–65)	68 (45–92) (*p* < 0.05)			
			LCW	42 (19–63)	63 (38–90) (*p* < 0.05)			
			Tegner (range)					
			MOW	3.2 (1.5–5)	4.3 (2.6–6) (*p* < 0.05)			
			LCW	3.1 (1–5.2) Definition of pre-op: unknown	3.9 (2.5–5.5) (*p* < 0.05)			
Study: Isolauri et al. [37], 1983, Finland Design: ret cohort study; FU 3 years (range 1–5) Population: Pts with unicompartmental knee OA and malalignment (*n* = 50: varus 32, valgus 18); age at operation 58 (range 33–77); sex 15 M (30%), 35 F (70%); BMI NS; Co: *n* = 26 (RA 1; HT 11; cardiac 8; diabetes 3; hyperthyroid 2; epilepsy 1)	HTO: LCW 32, MCW: 18 Fixation: Charnley’s compression device 16, plaster 34 (8 weeks)	Mobilization on crutches postoperative d1. Full weight bearing allowed after 3–4 weeks	–	–	–	Unknown Time to RTS: unknown	41%. Time to RTW: 5.5 months (2.5–11 months). Working capacity at FU: return to previous work 10 of 12 (83%). Trained for new occupation 2 of 12 (17%) Disabled on account of knee OA 13 (26%) Disabled on account other disease 4 (8%) Pension 21 (42%)	Adjusted for in analysis: obtained correction. Mentioned, not adjusted for: co; reasons other than HTO for no RTW
Study: Korovessis et al. [39], 1999, Greece Design: pro; FU 11 years (range 10–12) Population: Pts with medial compartment OA and varus malalignment who were employed in agriculture. Group I: *n* = 35; age 60 (range 49–74); sex 7 M (20%), 28 F (80%); BMI NS; Co NS. Group II: *n* = 28; age 65 (range 50–79); sex 7 M (25%), 21 F (75%); BMI NS; Co NS	Group I: Two-level “gap” osteotomy (Mittelmeier) Group II: LCW HTO Fixation: gap osteotomy: non-locking plate; LCW: AO buttress plate	Partial weight-bearing for 6–12 weeks	–	– Definition of pre-op: pre-surgery	–	Unknown	89% (in both groups). Time to RTW: 8–12 months	Mentioned, not adjusted for: age, pt motivation (“agricultural workers have to work until they are 80 years old”)
Study: Lerat et al. [48], 1993, France [French] Design: ret cs; FU 4 years (range 4–11) Population: Pts with chronic ACL deficiency associated with medial OA and varus malalignment (*n* = 49); age 37 (range 25–58); sex 39 M (80%), 10 F (20%); BMI NS; Co NS	Valgising HTO + ACL reconstruction Fixation: plate 20, staples 31	Removable splint for 4–6 weeks. Early mobilization with CPM. Weight bearing allowed after 2 months	Sports participation (pre-injury/pre-surgery, *n*(%))	*n* = 28	*n* = 28	48/63% RTS (%)	Unknown. Time to RTW: 5.1 months ± (range 3–18)	Mentioned, not adjusted for: surgeon’s advice
			Competition	10/5	2	20/40		
			Boxing	NS	1	NS		
			Tennis	NS	1	NS		
			Recreational sport	15/14	10	67/71		
				Definition of pre-op: pre-injury and pre-surgery		Time to RTS unknown		
Study: Minzlaff et al. [49], 2016, Germany Design: Cross-sectional; FU 6.9 years (range 2.5–9.8) Population: Pts with focal osteochondral defects of medial condyle and varus malalignment (*n* = 30); age 31 (range 19–39); sex NS; BMI 25 (range 21–32); Co NS; concomitant procedures: OATS 30	LCW HTO *n* = 16; MOW HTO *n* = 14 Fixation: LCW: non-locking L-plate; MOW: TomoFix	CPM for 6–8 weeks, ROM not restricted. 6 weeks non-weight bearing, increased with 20 kg/week. PT for 6–8 weeks. RTS (contact sports) allowed after osteotomy healing	Tegner	5 (2–7)	5 (4–7)	77%	Unknown	Adjusted for in analysis: age; defect size; number of previous surgeries. Mentioned, not adjusted for: donor-site morbidity
			Sports participation, *n*(%):			RTS (%)		
			Overall	30	23	77		
			Oarsmanship	1	1	100		
			Horseback riding	1	1	100		
			Martial arts	0	2	>100		
			Volleyball	1	2	>100		
			Basketball	1	0	0		
			Handball	0	1	>100		
			Soccer	7	6	86		
			Badminton	1	1	100		
			Table tennis	1	2	>100		
			Tennis singles	0	1	>100		
			Ice hockey	1	0	0		
			Snowboarding	3	4	>100		
			C-C skiing	3	4	>100		
			Downhill skiing	5	8	>100		
			Gymnastics	0	2	>100		
			Fitness training	9	10	>100		
			Swimming	10	11	>100		
			Mountain biking	5	9	>100		
			Cycling	15	17	>100		
			Climbing	1	0	0		
			Hiking	3	8	>100		
			Inline skating	3	2	67		
			Jogging	8	7	88		
			Nordic walking	2	3	>100		
				Definition of pre-op: lifetime and 1 year pre-surgery		Time to RTS: unknown		
Study: Nagel et al. [50], 1996, USA Design: ret; FU 8 years (range 2–14) Population: Pts with medial compartmental OA and varus malalignment (*n* = 34 [37 knees]). Group 1 (*n* = 12): preoperative Tegner ≤4. Group 2 (*n* = 22): preoperative Tegner ≥5. Age 49 (range 28–60); sex: 34 M (100%); BMI NS; Co NS	LCW HTO Fixation: above-the-knee cast 28, blade plate 8	NS	Sports participation, *n*(%):			RTS (%)	Unknown. 26/34 regularly performed manual labor (painting, laying tile, paneling, carpentry, gardening, construction work). Time to RTW unknown	Adjusted for in analysis: pre-op sports level (most predictive for RTS). Mentioned, not adjusted for: sex; surgeon’s advice
			Overall	–	25	–		
			Tennis	15	13	87		
			Downhill + C-C skiing	11	9	82		
			Jogging	14	10	71		
			Cycling	30	26	87		
			Tegner (range)					
			Group I (*n* = 12)	3.2 (2–4)	2.8 (1–4)			
			Group II (*n* = 22)	6.5 (5–8)	5.9 (2–8)			
				Definition of pre-op: unknown (presumably pre-surgery)		Time to RTS: unknown		
Study: Niemeyer et al. [51], 2008, Germany Design: pro; FU 2 years Population: Pts with medial compartment OA and varus malalignment (*n* = 43); age 47.3 ± 10.3 (range NS); sex: 37 M (86%) 6 F (14%); BMI 27.2 ± 3.5; Co NS; concomitant procedures: *n* = 37 (ACL reconstruction 1; microfracturing 24; partial meniscectomy 17; ACI 7)	MOW HTO Fixation: TomoFix	Pts were mobilized on postoperative d1. Weight bearing limited to 15 kg for 6 weeks, after which, full weight bearing was allowed in all cases	Pre-disease sports activity level, *n*(%)			68% regained predisease level of activity at 24 mo FU Time to RTS: unknown	Unknown	Adjusted for in analysis: smoking. Mentioned, not adjusted for: additional surgery; fixation type; pre-op sports level
			6 months	-	13 (30%)			
			12 months	-	25 (58%)			
			24 months	-	29 (68%)			
			Lysholm	5	78 ± 20 (p < 0.01)			
			IKDC (subjective)	40 (NS)	70 (NS) (p < 0.01)			
			IKDC (objective), *n*(%)					
			Normal	4 (9%)	19 (44%)			
			Nearly normal	16 (37%)	10 (23%)			
			Abnormal	15 (35%)	12 (28%)			
			Severely abnormal	8 (18%)	2 (5%)			
				Definition of pre-op: pre-symptomatic				
Study: Noyes et al. [33], 2000, USA Design: Pro cs; FU 4.5 years (range 2–12) Population: Pts with ACL deficiency and partial or complete lateral ligament deficiency and varus malalignment. Double varus: *n* = 23; age 30 (range 19–47); sex 21 M (91%), 2 F (9%); BMI NS. Triple varus: *n* = 18; age 28 (range 16–46); sex 11 M (61%), 7 F (39%); BMI NS. Co NS	LCW HTO Fixation: L-shaped internal plate	Long-leg brace for 8 weeks. Immediate ROM (0–90°). Toe-touch weight bearing for 3 weeks, gradually increased to full by wk 8–10. Quadriceps muscle isometric exercises, straight leg raises, patellar mobilization, and EMS	Sports participation, *n*(%):				>100%. Time to RTW unknown	Mentioned, not adjusted for: surgeon’s advice; non-homogenous population; staged surgery for complex cases
			Overall	14	27	>100%		
			Jumping, pivoting, cutting	2	3	>100%		
			Running, twisting, turning	9	4	44%		
			Low impact (swimming, biking)	3	24	>100%		
			No sports	27	10	37%		
			Employment, *n*(%)			Time to RTS unknown	RTW (%)	
			Overall	23	34		>100	
			Light	11	20		>100	
			Moderate	9	10		>100	
			Very heavy	3	4		>100	
			Student/homemaker	9	4		–	
			Disabled (because of knee condition)	9	3		33	
				Definition of pre-op: pre-surgery				
Study: Saier et al. [52], 2015, Germany Design: pro cs; FU 2 years Population: Pts aged <65 with medial compartment OA and varus malalignment (*n* = 64); age 45.5 (range 20–63); sex 46 M (74%), 18 F (26%); BMI 26.6 (range 19–35); Co NS	MOW HTO (biplanar) Fixation: TomoFix plate, Peek power plate	Immediate FROM. Partial weight bearing for 2 weeks, increased by 20 kg/wk until full weight bearing. RTS allowed after 3 months and contact sports after osseous consolidation	–	– Definition of pre-op: pre-surgery	–	Unknown	93% (45/50). 90% without symptoms; 3% with impairment; 7% did not RTW due to knee symptoms. Time to RTW: 5.2 mo (range 1.5–24)	Adjusted for in analysis: psychological distress. Mentioned, not adjusted for: fixation type, surgeon’s advice
Study: Salzmann et al. [53], 2009, Germany Design: Cross-sectional; FU 3 years (range 1.2–7) Population: Pts aged <65 with medial compartment OA and varus malalignment (*n* = 65); age 41.2 (range 19–65); sex 51 M (78%), 14 F (22%); BMI 21 (range 20–34); Co NS; concomitant procedures: *n* = 9 (partial meniscectomy 6, OATS 2, notchplasty 1)	MOW HTO (biplanar) Fixation: TomoFix plate	Partial weight bearing (15 kg) for 4 weeks. Weight-bearing gradually increased from week 4–6 and full weight bearing after 6–8 weeks	Sports activity (lifetime/pre-operative, *n*(%))			95% RTS (%)	Unknown	Adjusted for in analysis: age, ASA, BMI, concomitant procedures, correction angle, sex, KL score, satisfaction. (None of these factors were correlated with sports participation)
			Overall	62/57	59	95/>100		
			Cycling	47/43	46	99/>100		
			Downhill skiing	35/18	18	51/100		
			Swimming	33/42	30	92/71		
			Hiking	29/17	20	68/>100		
			Fitness	27/13	17	63/>100		
			Mountain biking	19/13	14	70/>100		
			C-C skiing	19/7	5	28/71		
			Tennis singles	16/3	2	13/67		
			Volleyball	15/3	3	22/100		
			Inline skating	15/8	6	39/75		
						Time to RTS: unknown		
			Tegner (range)	4.9 (1–10)	4.3 (2–9) (*p* < 0.05)			
			Lysholm (range)	42 (7–90)	70 (22–95) (*p* < 0.01)			
				Definition of pre-op: during lifetime and pre-surgery				
Study: Saragaglia et al. [35], 2014, France Design: ret; FU 5.8 years (range 5–9) Population: Pts with medial compartment OA and varus malalignment (*n* = 83); age 50.4 (range 32–67); sex 56 M (68%), 27 F (32%); BMI 27.5 ± 4.7. Previous surgery: medial meniscectomy 23, ACL reconstruction 10; Co: 16% medical conditions that could hinder RTS	MOW HTO (*n* = 62) MOW HTO + LCW DFO (double osteotomy): *n* = 21 Fixation NS	NS	Sports participation, *n(%)*			>100% RTS (%)	Unknown	Adjusted for in analysis: age, BMI, sex, type of osteotomy, motivation, pre-existent sports level. Mentioned, not adjusted for: Co, effect of double osteotomy, reasons for non-RTS
			Overall	66	71	>100		
			Cycling	28	26	93		
			Power walking	22	26	>100		
			Downhill skiing	22	14	64		
			Running	20	17	85		
			Hiking	12	6	50		
			Swimming	9	13	>100		
			Tennis	5	5	100		
			Football	4	1	25		
			C-C skiing	4	1	25		
			Ski touring	3	3	100		
			Gymnastics	3	2	67		
			Gardening	2	3	>100		
			Climbing	2	2	100		
			Windsurfing	2	2	100		
			Mountain bike	2	1	50		
			Bodybuilding	1	2	>100		
			Golf	1	1	100		
			Handball	1	1	100		
			Bowls	1	1	100		
			Hunting	1	1	100		
			Squash	1	1	100		
			Diving	1	1	100		
			Volleyball	1	0	0		
			Rugby	1	1	100		
			Basketball	1	0	0		
			Lysholm (range)	63 (30–100)	91 (55–100) (*p* < 0.001)	66 (80%) returned to same sporting level as before onset of OA. Time to RTS unknown		
			Tegner (range)	4.5 (range NS)	4.1 (range NS) (*p* = 0.07)			
			UCLA (range)	7.1 (range NS)	6.6 (range NS) (*p* = 0.09)			
				Definition of pre-op: pre-symptomatic				
Study: Schröter et al. [54], 2013, Germany Design: ret; FU 6.4 ± 1.6 years (range NS) Population: Pts with medial compartment OA and varus malalignment who were employed at time of surgery (*n* = 32); age 47 ± 9; sex 22 M (69%), 10 F (31%); BMI 28.6 ± 4.7; Co NS	MOW HTO Fixation: LC-DCP plate	No brace or cast. 20-kg partial weight bearing for 6 weeks, full after 6–8 weeks. Active physiotherapy started after removal of drains	Lysholm (±SD)	62.5 (±17.5)	81.7 (±12.7) (*p* < 0.01)	Unknown	Unknown. Time to RTW: 87 days (14–450). Time to RTW for each REFA category: 0 = 42 days (14–150) 1 = 90 days (40–180) 2 = 120 days (28–450) 3 = 66 days (60–300) 4 = 120 days (120–120) 3 (9%) pts changed employment to occupation with lower workload	Adjusted for in analysis: workload. Mentioned, not adjusted for: fixation type, rehabilitation protocol, surgeon’s advice
			Tegner (range)	3 (1–5)	4 (1–8) (*p* = NS)			
			REFA work (physical strain, *n*(%)					
			0 (without)	7 (22)	8 (25)			
			1 (small)	11 (34)	11 (34)			
			2 (moderate)	8 (25)	9 (28)			
			3 (hard)	5 (16)	3 (9)			
			4 (most heavy)	1 (3)	1 (3)			
Study: Waterman et al. [55], 2015, USA Design: ret; FU 4.0 years (range 2–8) Population: Active US duty service members undergoing HTO for coronal plane malalignment and/or intraarticular pathology (*n* = 181 [202 HTOs]); age 35.7 (range 15–55); sex 169 M (93%), 12 F (7%); BMI NS; Co NS. Concomitant procedures: *n* = 87 (meniscal 48, chondral 40, ligamentous 48)	MOW HTO Fixation: plate fixation (*n* = 171); external/ring fixation (*n* = 12); unspecified (*n* = 19)	NS	Combat deployment record, *n*(%)	34 (19)	15 (8.3)	Unknown	72% returned to military duty, 43% without limitations. 8.3% successfully completed postoperative combat deployment. 41% had minor permanent activity limitations	Adjusted for in analysis: age, complications, concomitant procedures, sex, smoking. Mentioned, not adjusted for: selected (military) population, surgeon’s advice
				Definition of pre-op: pre-surgery				
Study: Williams et al. [56], 2003, USA Design: ret; FU 3.8 years (range 2.0–8.8) Population: Pts with chronic ACL deficiency, medial compartment OA and varus malalignment (*n* = 25); age 35 (range 26–46); sex 18 M (72%) 7 F (28%); BMI NS; Co NS	LCW HTO (*n* = 12) LCW HTO + ACL reconstruction (*n* = 13) Fixation: two staples	Hinged knee brace. Non-weight bearing for minimum 4 weeks	Sports participation, *n(%)*			RTS (%)	Unknown	Adjusted for in analysis: concomitant procedures (ACL
			Overall	13	25	>100		
			Competitive sports	2	4	>100		
			Recreational sports	12	19	>100		
			Unable to participate in sports activities	11	2	–		
			Lysholm (range)					
			Group 1	46.8 (19–64)	76.3 (57–100) (*p* < 0.05)			
			Group 2	47.0 (14–73)	80.9 (56–95) (*p* < 0.05)			
			Tegner (range)					
			Group 1	3.8 (1–7)	4.9 (3–7) (*p* < 0.02)			
			Group 2	3.6 (1–7)	4.7 (3–8) (*p* < 0.02)			
				Definition of pre-op: immediately prior to surgery				
Study: Yim et al. [57], 2013, South Korea Design: cross-sectional; FU 3.6 years (range 3–4) Population: Pts with medial compartment OA and varus malalignment (*n* = 58); age 58.3 (range 43–65); sex 7 M (12%), 51 F (88%); BMI NS; Co NS	MOW HTO Fixation: two Aescula wedge plates	ROM exercises, patellar mobilization, and straight-leg raises from postoperative d1. Partial weight bearing after 6 weeks, full weight bearing with a crutch after 8–12 weeks	Tegner	3.1 ± 1.1	2.5 ± 1.2 (*p* = NS)	78%. Time to RTS unknown	Unknown	Mentioned, not adjusted for: age, selected population (rural areas)
			Lysholm	62.4 ± 9.5	89.6 ± 8.7 (*p* = NS)			
			Participation in ≥1 low-impact activities, *n*(%):					
			0 activities	8	19			
			≥1 activities	50 Definition of pre-op: pre-surgery	39			

*ACI* autologous chondrocyte implantation, *ACL* anterior cruciate ligament, *ADL* activities of daily living, *AO* Arbeitsgemeinschaft für Osteosynthesefragen, *ASA* American Society of Anesthesiologists, *BMI* body mass index, *BPTB* bone patellar tendon bone, *C-C* cross-country, *Co* co-morbidities, *CPM* continuous passive motion, *cs* case series, *d* day, *DFO* distal femoral osteotomy, *DH* downhill, *EMS* electronic muscle stimulation, *F* female, *FROM* free range of motion, *FU* follow-up, *HT* hypertension, *HTO* high tibial osteotomy, *IKDC* International Knee Documentation Committee, *KL* Kellgren-Lawrence, *LAD* ligament augmentation device, *LC-DCP* limited-contact dynamic compression plate, *LCW* lateral closing wedge, *LET* lateral extra-articular tenodesis, *M* male, *mc* multicenter, *MOW* medial opening wedge, *NS* not stated, *n.s*. not significant, *OA* osteoarthritis, *OATS* osteochondral autograft transplant system, *pre-op* preoperative, *pro* prospective, *PT* physiotherapy, *pts* patients, *RA* rheumatoid arthritis, *REFA* Reichsausschuss für Arbeitszeitermittlung, *ret* retrospective, *ROM* range of motion, *RTS* return to sports, *RTW* return to work, *SD* standard deviation, *TKA* total knee arthroplasty, *UCLA* University of California, Los Angeles

^a^Data are mean ± SD except otherwise indicated; age is presented in years unless otherwise indicated; BMI is presented in kg/m^2^

### Data Extraction

One author (AH) extracted data from all selected original studies, and this was independently repeated by one other author (SW). Disagreements were resolved by discussion. The authors used a standardized data extraction form that included the following: (1) study information: author, year, country, and reference number; (2) study design and follow-up; (3) information about study population: cohort, population size, sex, age, body mass index (BMI), comorbidities; (4) description of rehabilitation protocols used; (5) definition of outcome measures; (6) preoperative activity and definition (e.g., pre-symptomatic or at time of surgery); (7) postoperative activity; (8) RTS and RTW percentages and time to RTS and RTW; (9) confounding factors taken into account for RTS and RTW, such as age, sex, BMI, restricting comorbidities, complications, preoperative sports or work level, surgeon advice, or psychosocial factors. Authors were contacted if data were missing or only available in graphs. If this information was not provided, available data were read off the graphs.

### Pooling Data

Data were pooled from the studies that described pre- and/or postoperative participation in specific types of sports and categorised into low-, intermediate-, or high-impact sports according to the levels of impact on the knee joint (ESM Appendix S3). This classification complies with Vail et al. [31] and is supported by a biomechanical study from Kuster et al. [32], which considered both peak loads and flexion angles of the knee. We calculated pooled RTS percentages by comparing pooled pre- and postoperative sports participation data. In addition, we compared percentages for RTS to the preoperative level and the pre-symptomatic level. We also pooled RTW data for studies that provided pre- and postoperative work data.

## Results

### Literature Search

**Fig. 1:**
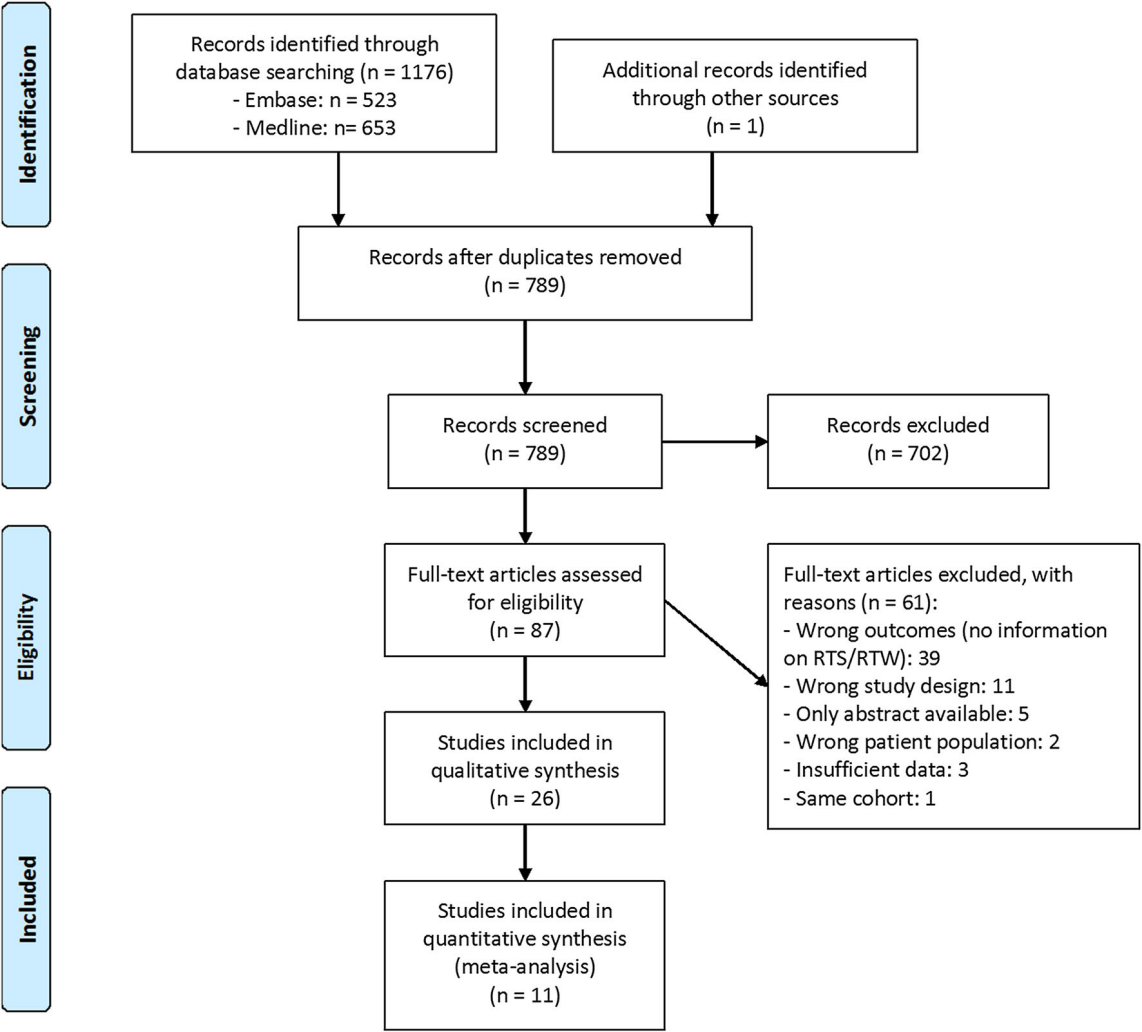
PRISMA flow diagram

Figure 1 presents the PRISMA flowchart for our search strategy. Our primary search retrieved 1176 potentially relevant citations. After deleting 387 duplicates, we applied our inclusion criteria to the titles and abstracts of 789 articles. Of the 789 screened articles, disagreement occurred in 45 cases (6%), which were all resolved by discussion. This selection yielded 87 potentially relevant full-text articles, which were then reviewed. For the full-text screening, disagreement occurred in four (5%) cases, which were resolved by discussion. We subsequently excluded 61 articles for various reasons (Fig. 1). Noyes et al. [33] published two studies involving the same cohort, so we only included the study with the longest follow-up. We performed reference screening and forward citation tracking on the remaining articles, which yielded one additional article [34]. Finally, 26 articles were included.

### Study Characteristics

#### Demographic Data

Table 1 presents the results of the data extraction. Studies were published between 1983 and 2016, and all the included studies were observational, with four cross-sectional studies, five prospective cohort studies, 14 retrospective cohort studies, and three retrospective case series. One study was performed in Brazil, one in Finland, five in France, nine in Germany, one in Greece, one in Italy, one in South Korea, one in Sweden, one in Switzerland, and five in the USA. The majority of studies were written in English (*n* = 24), one was in French, and one was in Italian. The total number of included patients was 1321 (range 6–181), sex was specified in 24 studies (1251 patients; 857 [69%] male). Mean age ranged from 27 to 62 years (range 14–80). The mean duration of follow-up was 4.8 years (range 1.8–11.0). Patients’ BMI was specified in 12 studies, with mean BMI varying from 21 to 30 kg/m^2^. Three of 26 studies included information on comorbidities.

#### Surgical Technique

Nine studies included only medial open-wedge (MOW) HTO, four only lateral closing-wedge (LCW) HTO, six both MOW HTO and LCW HTO, one MOW HTO and MOW HTO + LCW DFO [35], one both MOW HTO and lateral opening-wedge (LOW) DFO [36], one both LCW and medial closing-wedge (MCW) [37], and one LOW DFO [38]. One study reported the use of LCW HTO and a ‘Mittelmeier’ HTO, which was not further specified [39], one study performed MOW HTO with external fixation (hemicallotasis technique) [40], and one study only mentioned the use of both varising and valgising HTO, but the type was not further specified [41]. For fixation, 20 studies used plate fixation, with six studies using the TomoFix plate, two studies using the Peak-carbon plate, one study using the Puddu plate, and 11 studies using other types of plates (for more details, see Table 1). Seven studies used staples, two studies used external fixators, two studies used plaster casts, and three studies did not describe their fixation method. Concomitant surgery was performed in eight studies, with anterior cruciate ligament (ACL) reconstruction performed in five studies, autologous chondrocyte implantation performed in two studies, and meniscal allograft transplantations performed in one study (Table 1).

### Methodological Quality

Overall, 7 of 26 studies scored a low risk of bias, ten studies scored a moderate risk of bias, and nine studies scored a high risk of bias. The lowest risk of bias was found for the prognostic factor domain, describing the type of osteotomy performed and any additional surgery, for which no study scored a high risk of bias. The highest risk of bias was found for the confounding factors (e.g., patient-related factors, surgeons’ advice, rehabilitation), with 17 studies scoring a high risk and only four studies scoring a low risk of bias. Table 2 summarizes the methodological assessment for the risk of bias.

**Table 2:** Methodological assessment according to six domains of potential bias (QUIPS)

Study (*n* = 26)	Study participation	Study attrition	Prognostic factor	Outcome	Confounding factors	Analysis	Overall risk of bias^a^
Ampollini et al. [42]	Moderate	Low	Low	Moderate	High	Moderate	Moderate
Bode et al. [25]	Low	Low	Low	Low	High	Low	Moderate
Bonnin et al. [43]	Moderate	High	Moderate	Low	High	Low	High
Boss et al. [44]	Moderate	Low	Low	High	High	Low	High
Boussaton et al. [41]	Moderate	Low	Moderate	High	High	Moderate	High
Cotic et al. [45]	Low	Low	Low	Low	Moderate	Low	Low
Dahl et al. [40]	Low	Low	Low	Low	Moderate	Low	Low
De Carvalho et al. [38]	Low	Moderate	Low	Moderate	High	Low	Moderate
Dejour et al. [46]	Moderate	High	Low	Low	High	High	High
Faschingbauer et al. [47]	Low	Moderate	Low	Low	Moderate	Low	Low
Gomoll et al. [36]	Low	Low	Low	Low	High	High	High
Hoell et al. [34]	Moderate	Moderate	Low	Low	High	High	High
Isolauri et al. [37]	High	High	Moderate	High	High	High	High
Korovessis et al. [39]	Low	Moderate	Moderate	Moderate	High	Low	Moderate
Lerat et al. [48]	High	High	Moderate	Moderate	High	Low	High
Minzlaff et al. [49]	Low	Low	Low	Moderate	Low	Low	Low
Nagel et al. [50]	High	High	Low	Low	Moderate	Moderate	High
Niemeyer et al. [51]	Low	Low	Low	Low	High	Low	Moderate
Noyes et al. [33]	Moderate	Low	Low	Moderate	High	Low	Moderate
Saier et al. [52]	Low	Moderate	Low	Low	Low	Low	Low
Salzmann et al. [53]	Moderate	Moderate	Low	Moderate	High	Moderate	Moderate
Saragaglia et al. [35]	High	Moderate	Low	Moderate	Low	Low	Moderate
Schröter et al. [54]	Low	Moderate	Low	Low	High	Low	Moderate
Waterman et al. [55]	Low	Low	Moderate	Low	Low	Moderate	Low
Williams et al. [56]	Moderate	Moderate	Low	Moderate	High	Moderate	Moderate
Yim et al. [57]	Low	Low	Low	Moderate	Moderate	Low	Low

*QUIPS* Quality in Prognosis Studies

^a^We considered a study to be of low risk of bias when the methodological risk of bias was rated as low or moderate on all of the six domains, with at least four rated as low. A study was scored as high risk of bias if two or more of the domains were scored as high

### Return to Sport

In total, 19 of 26 studies reported the percentage of patients returning to different types of sport activities. Mean RTS percentages varied from 48 to >100%, with >100% indicating that more patients participated in sports activities postoperatively than preoperatively. A definition of preoperative sports participation was provided in 16 of 26 studies. Seven studies describing the preoperative sports level as the moment prior to surgery (pre-surgery level) found RTS varied from 66 to >100%. Nine studies describing the preoperative sports level as the moment before the onset of knee symptoms (pre-symptomatic level) found that 68–100% could return to this level. Of the studies with low risk of bias, five provided RTS percentages: 63% (at 10 years), 78, 92, 100 and >100% (more patients participated in sports postoperatively than preoperatively). None of the included studies reported on the timing of RTS.

Data could be pooled for 16 studies that reported exact numbers of patients participating in sports pre- and/or postoperatively. Overall, RTS was 94%, but this depended on how the preoperative sports level was defined (Table 3). Seven studies used the pre-surgery level and found an average RTS of >100%. Nine studies used the pre-symptomatic level and found an average RTS of 85%. For the studies scoring a low risk of bias, three studies used the pre-surgery level and found an average RTS of 89%. Two studies used the pre-symptomatic level and found an average RTS of 78%. In total, 11 studies reported specific numbers of sports that were practiced pre- and postoperatively (Table 4). Preoperatively, 453 patients practiced an average of 1.9 sports, including 47% low-impact sports, 35% intermediate-impact sports and 18% high-impact sports. Postoperatively, 592 patients practiced an average of 1.9 sports, including 58% low-impact sports, 32% intermediate-impact sports and 10% high-impact sports. Five of 11 pooled studies were rated as having a low risk of bias. In these studies, 204 patients practiced an average of 1.9 sports preoperatively, including 55% low-impact sports, 32% intermediate-impact sports and 12% high-impact sports. Postoperatively, 204 patients practiced an average of 1.9 sports, including 56% low-impact sports, 35% intermediate-impact sports and 9% high-impact sports.

**Table 3:** Pooled data for numbers of patients participating in any sport pre- and postoperatively

Preoperative reference for RTS	No. of pts participating in any sport preoperatively	No. of pts participating in any sport postoperatively	RTS (%)
Overall (16 studies)	463	434	94
Pre-surgery status as reference for RTS (7 studies)	150	167	111
Pre-symptomatic status as reference for RTS (9 studies)	313	267	85
Low risk of bias studies (5 studies)	181	149	82

*pts* patients, *RTS* return to sport

**Table 4:** Pooled data for pre- and postoperative sports participation for different types of sports impact

Impact	Sports participation preoperatively (*n* = 10 studies)			Sports participation postoperatively (*n* = 11 studies)		
	Sports (*n*)	Patients (*n*)	Average sports/patient, *n* (%)	Sports (*n*)	Patients (*n*)	Average sports/patient, *n* (%)
Low (e.g. cycling, swimming, golfing)	413	453	0.91 (47)	658	592	1.11 (58)
Intermediate (e.g. hiking, downhill skiing)	303	453	0.67 (35)	369	592	0.62 (32)
High (e.g. tennis, running, ball sports)	159	453	0.35 (18)	109	592	0.18 (10)
Total	875	453	1.93	1136	592	1.92

### Return to Work

In total, 11 of 26 studies reported on the possibility of RTW after HTO (Table 1). Mean RTW varied from 41 to >100%, with >100% indicating that more patients were working postoperatively than preoperatively. For the studies with a low risk of bias, RTW rates were 72, 84, 93 and 94%. One study investigated a military population with a very high workload and found that 72% could RTW [55]. Another study investigated an agricultural population with a high workload and found that 86% could RTW [39]. Four studies reported on the timing of RTW, which varied from 9.7 to 22.1 weeks. One additional study reported that 89% of an homogeneous group of agricultural workers had returned to work after 8–12 months, but did not specify the exact timing [39]. Two studies found timing of RTW was significantly dependent on the workload, which was assessed using the REFA workload classification [25, 54]. Duration of inability to work varied from 6 and 10 weeks for REFA grade 0 (lowest workload) to 17 and 22 weeks for REFA 4 (heaviest physical strain) (*p* < 0.05). In line with these findings, Faschingbauer et al. [47] found that workers with the highest workload returned after 19.1 weeks and those with the lowest workload returned after 11.8 weeks, although this difference was not statistically significant. In terms of working capacity at follow-up, 72–100% of patients returned to the same or a higher workload. Finally, one study investigating RTW after DFO found that 89% of patients could RTW [38]. The duration of inability to work was not mentioned.

Data could be pooled for seven studies, including two with a low risk of bias, which reported exact numbers of patients working pre- and postoperatively. Overall, 85% of patients could RTW (Table 5). In studies with a low risk of bias, 80% could RTW. Six studies described the duration of inability to work. On average, patients were unable to work for 16 weeks (Table 5). Two studies with a low risk of bias reported that patients were unable to work for an average of 19 weeks. This included the study by Saier et al. [52], who found that, overall, patients were unable to work for 21 weeks. Separate analysis showed that patients with a concomitant mental disorder could RTW after an average of 36 weeks compared with 16 weeks in the mentally healthy group.

**Table 5:** Pooled data for return to work and average duration of inability to work

Study (*n* = 7)	Number of working patients			Time to RTW		
	Preoperative (*n*)	Postoperative (*n*)	RTW (%)	Study (*n* = 6)	Patients (*n*)	Inability to work (weeks)
Dahl et al. [40]	43	38	88	Bode et al. [25]	40	13.5
De Carvalho et al. [38]	26	23	88	Faschingbauer et al. [47]	40	16.7
Faschingbauer et al. [47]	43	40	93	Hoell^a^ (ow) et al. [34]	40	13.9
Korovessis et al. [39]	63	54	86	Hoell^a^ (cw) et al. [34]	51	13.6
Noyes et al. [33]	23	34	148	Lerat et al. [48]	49	20
Saier et al. [52]	50	45	90	Saier et al. [52]	64	20.8
Waterman et al. [55]	181	130	72	Schröter et al. [54]	32	12.4
Total	429	364	85	Total	276	16.3

*RTW* return to work, *OW* opening-wedge, *CW* closing-wedge, *HTO* high tibial osteotomy

^a^Hoell et al. reported separate duration of inability to work after opening-wedge HTO and closing-wedge HTO

### Secondary Outcome Measures of Physical Activity

The Tegner score, Lysholm score, UCLA score, and IKDC score were described as secondary outcome measures for physical activity. IKDC scores (0–100) were used in three studies. Gomoll et al. [36] and Niemeyer et al. [51] described median preoperative scores of 26 and 40 and median postoperative scores of 63 and 70, respectively. Boussaton and Potel [41] described a median postoperative IKDC score of 94 (range 86–99). The Lysholm score was described in 12 studies, with median preoperative scores ranging from 5 to 63 and median postoperative scores ranging from 63 to 91. The Tegner score was described in 11 studies, with median preoperative scores ranging from 3.1 to 6.5 and median postoperative scores ranging from 2.5 to 5.9. The UCLA score was described in one study, with a median preoperative score of 7.1 and postoperative score of 6.6 [35].

### Confounders

We scored whether studies mentioned possible confounders, and whether analyses were adjusted for these confounders. Possible confounders that could influence RTS and/or RTW were mentioned in 25 of 26 studies, but only 15 studies adjusted for one or more confounders in the analysis. Age was mentioned as a possible confounder in 11 studies, and three studies adjusted for it. Minzlaff et al. [49] found that younger patients reached a higher frequency of post-operative sports. In contrast, Salzmann et al. [53] and Saragaglia et al. [35] found age had no influence on RTS. BMI was mentioned as a possible confounder in four studies. Two studies adjusted for BMI but found no influence on RTS. Four studies mentioned sex as a confounder, and three studies adjusted for it but found no effect on RTS. Three studies mentioned comorbidities as a possible confounder. Salzmann et al. [53] adjusted for comorbidities using the American Society of Anesthesiologists classification but found no correlation with RTS. Saragaglia et al. [35] specifically mentioned reasons for patients who could not RTS. Of 12 patients, four had medical contraindications, three had severe intractability, and five indicated that the knee was solely responsible for the inability to RTS. Four studies mentioned concomitant procedures as a possible confounder. Salzmann et al. [53] found no effect of concomitant procedures on RTS, whereas Waterman et al. [55] found that concomitant procedures increased the risk of failure. The influence of patient motivation was mentioned in four studies. Bonnin et al. [43] found motivation to be strongly correlated to RTS, whereas Saragaglia et al. [35] found no correlation. The preoperative sports level was mentioned as a confounder in six studies. Nagel et al. [50] found preoperative sports level to be the most predictive factor for RTS, whereas Saragaglia et al. [35] found no correlation. The influence of the surgeons’ advice on RTS was mentioned in nine studies. Most surgeons in these studies advised their patients that RTS was not the goal of surgery and tried to moderate their patients’ sporting ambitions. Faschingbauer et al. [47] and Noyes et al. [33] discouraged participation in high-impact activities such as soccer and tennis. The rehabilitation protocol was mentioned in 19 of 26 studies, but the description was often very brief, only including information about the first phase of rehabilitation, concerning range of motion (ROM) and weight-bearing advice. Five studies described their RTS advice in detail. Three studies [36, 44, 52] advised a return to activities of daily life and low-impact sports after 3 months and a return to more demanding activities and contact sports after 6–12 months. Two studies [38, 49] allowed full RTS, including contact sports, after radiologically confirmed healing of the osteotomy.

Finally, three studies adjusted for the effect of workload on RTW: two of these [25, 54] found that higher workloads resulted in longer inability to work, but one study [47] found no significant difference in RTW between high and low workloads. Only one study [34] compared RTW for different types of HTO; it found no significant difference in time to RTW between open- and closed-wedge HTO.

## Discussion

Our systematic review showed that a large percentage of patients were able to RTS activities and RTW after osteotomies around the knee. Concerning sports activities, 66 to >100%, with >100% indicating more patients participated in sports postoperatively than preoperatively, of patients could RTS. An overall trend was observed towards participation in lower-impact activities after surgery. The diversity in RTS percentages was mostly caused by the different definitions used for the preoperative reference point for sports participation. Remarkably, none of the included studies reported on the timing of RTS. Concerning RTW, 41 to >100% of patients could RTW and 72–100% of patients could return to the same or a higher workload. The duration of inability to work varied from 10 to 22 weeks.

### Return to Sport

The meta-analysis showed that overall, 94% of patients could RTS, and 85% returned to their pre-symptomatic sports level after knee osteotomies. In a recent review on RTS and RTW after HTO, Ekhtiari et al. [58] found that 87% could RTS. However, the authors did not take into account the definition of preoperative sports participation, and our review showed that different definitions resulted in considerable variance in RTS percentages. Moreover, Ekhtiari et al. [58] only evaluated results of RTS and RTW after HTO, described in ten studies, including 250 patients, whereas we reviewed results after any osteotomy around the knee and found 16 studies, including 463 patients. Lastly, the indication for HTO was knee OA in almost all studies in their review. We observed that osteotomies around the knee are also increasingly performed for other indications, such as in addition to ligament reconstruction or articular cartilage restoration procedures. Such patients are often younger and thus more likely to wish to return to more demanding activities. For these patients in particular, it is imperative to know whether it is possible to RTS and RTW.

In a review of RTS after KA, Witjes et al. [6] found that 36–89% could RTS after total KA (TKA), and 74 to >100% could RTS following unicondylar KA (UKA). Postoperatively, patients undergoing TKA were engaged in an average of 1.0 sports, including 87% low-impact sports, 9% intermediate-impact sports, and 4% high-impact sports. Patients undergoing UKA were engaged in an average of 1.5 sports, including 77% low-impact sports, 19% intermediate-impact sports, and 4% high-impact sports. The present study demonstrates that patients participated in an average of 1.9 sports postoperatively, including 58% low-impact sports, 32% intermediate-impact sports, and 10% high-impact sports. Thus, on average, patients undergoing knee osteotomies returned to more sports than did patients undergoing KA. A shift to participation in lower-impact sports activities was observed in all three groups, but high-impact sports were performed more often after knee osteotomy than after KA. Thus, the possibility of returning to high-impact sports appears most likely after knee osteotomies and is also possible, though less likely, after UKA. In contrast, participation in high-impact sports after TKA is most unlikely. However, these findings could, at least in part, be explained by the generally younger age and less severe grades of knee OA in patients undergoing knee osteotomy compared with those undergoing KA.

#### Factors Influencing Return to Sport

The existing evidence on factors that influence RTS after knee osteotomy is ambiguous. Nagel et al. [50] found that the most predictive factor for RTS after HTO was the patient’s preoperative sporting level. Patient motivation appears to be another important factor. Mancuso et al. [59] found that only 30% of patients undergoing TKA expressed motivation to RTS, whereas Saragaglia et al. [35] found that 71% of patients undergoing HTO were motivated to RTS but that neither the motivation nor the pre-existent sport level was related to greater RTS. In contrast, Bonnin et al. [43] found a correlation between patient motivation and activity level, with motivated patients being more active postoperatively. These contrasting findings may be explained by the nature of the practiced sports. Despite high motivation, a return to high-impact sports is more difficult than a return to low-impact sports. Comorbidities that could possibly hinder patients in their RTS were only described in 3 of 26 studies. One study [35] found that 12 of 83 patients could not RTS because of comorbidities, and knee symptoms were solely responsible for the inability to RTS in five patients. Thus, we cannot rule out that specific medical conditions unrelated to the knee surgery had a negative influence on the number of patients that could RTS and RTW in other studies.

Our results confirm that, when assessing RTS, it is very important to use a clear definition of the preoperative sports level (e.g., preoperative, pre-symptomatic), as previously stated by Witjes et al. [6]. Remarkably, only 18 studies reported their definition, and only nine studies used the pre-symptomatic sports level to calculate RTS percentages. A return to pre-surgery sports level was possible in >100%, whereas a return to the pre-symptomatic level was possible in only 85%. We believe that the pre-symptomatic level is most relevant for young, active patients, since it is conceivable that this patient population in particular expects to return to the activities they performed before the onset of knee symptoms.

Finally, evidence on the return to professional or competitive levels of sports after knee osteotomies is sparse. A French study by Boussaton and Potel [41] followed six professional rugby players who all successfully returned to play, with follow-up varying from 1 to 10 years. Faschingbauer et al. [47] included four competitive-level athletes: two football players, one rugby player, and one squash player. Only one athlete, the rugby player, could return to competitive sport. In the study by Williams et al. [56], two patients participated in (unspecified) competitive sports preoperatively, whereas four patients were participating in competitive sports at a mean follow-up of 3.8 years. Lerat et al. [48] found that two of ten patients could return to competitive boxing and tennis, respectively. We found one other review describing two cases of National Football League players who successfully returned to play after HTO [26]. Still, the authors highlighted that, even in elite athletes, the goal of HTO is not resumption of competition but rather to allow daily and recreational-level activities. This consideration is in line with the surgeons’ advice that was described in nine of the studies included in this review. However, even without taking into account the effect of possibly discouraging advice from surgeons, our results show that a reasonable number of patients are able to successfully return to high-impact sports activities. Therefore, we believe that a return to competitive sports should not be ruled out in advance. As indicated, native knee structures are spared in knee osteotomies, without any risk of wear to a prosthesis. Thus, when full consolidation of the osteotomy is achieved, a return to competitive sports may be attempted. However, this also depends on the original indication for the osteotomy. Expectations of RTS may need to be tempered based on the indication.

### Return to Work

This review is the first to systematically assess the possibility of RTW after all types of knee osteotomies. We found that 364 of 429 (85%) patients could RTW and that the mean duration of their inability to work was 16.3 weeks. This is in line with the aforementioned review by Ekhtiari et al. [58], who found 310 of 367 (85%) patients could RTW. Based on existing studies, we cannot draw definite conclusions on the possibility of returning to the same or higher workloads. However, our findings do indicate that a RTW with high workloads (e.g., military service, work with heavy physical strain) is less likely than a RTW with low workloads.

#### Factors Influencing Return to Work

Our study is the first to describe factors influencing RTW after knee osteotomies. Such factors have been described before in patients undergoing KA and included a job with high physical demands on the knee, preoperative sick leave, and patient movement restrictions [60–62]. It seems reasonable that patients with physically demanding jobs need more time to RTW. Of the three studies we included that adjusted for workload, two found that higher workloads resulted in significantly longer inability to work [25, 54], but one study did not find this association [47]. Unfortunately, data on preoperative sick leave were not available for any of the included studies. Thus, more studies with larger patient groups are needed to clarify the relationship between these factors and RTW after knee osteotomy. Finally, the influence of movement restrictions could be partly compared between studies using the weight-bearing advice, which may influence the possibility of RTW. Immediate weight-bearing can allow for an earlier return to activities, including work. Recently, Lansdaal et al. [63] showed that immediate full weight-bearing compared with delayed full weight-bearing (2 months) after HTO with TomoFix plate fixation was safe and did not compromise functional outcome. The use of angle-stable fixation plates, such as the TomoFix plate, offers superior initial stability compared with other plates, and immediate weight-bearing is possible with this type of plate fixation [64]. Of six studies reporting on time to RTW, three used the TomoFix plate, one used the Association for the Study of Internal Fixation (AO) L-plate, one used the Puddu plate and/or staples, and one used an unspecified plate and/or staples. Only Saier et al. [52] and Faschingbauer et al. [47] reported the use of an early weight-bearing protocol after 2 weeks, and both studies used the TomoFix plate for fixation. Interestingly, the average time to RTW in the study by Saier et al. [52] was the longest of all included studies (21 weeks), whereas Faschingbauer et al. [47] reported an average of 17 weeks. The other studies reported 6–8 weeks of partial weight-bearing and found an inability to work of 12–20 weeks. Based on this evidence, we therefore cannot confirm or reject the hypothesis that using plates that allow early weight-bearing results in earlier RTW. Saier et al. [52] attributed their findings of a late RTW to the presence of mental disorder in the included patients, because separate analysis showed that patients with mental disorder took considerably longer to RTW than mentally healthy patients (36 vs. 16 weeks, respectively, on average). This emphasizes the importance of recognizing another important confounder, namely mental disorders, a known risk factor for worse outcome after knee surgery [65].

### Strengths and Limitations

One strength of the present systematic review is that we included all osteotomies around the knee and studies of all indications for osteotomies. Waterman et al. [55] observed that concomitant chondral restoration, meniscal and ligamentous procedures were performed in nearly half of 181 HTOs in a young military population. We believe that the use of osteotomies as an adjunct to reconstructive knee procedures in young, highly active patients will continue to increase. Therefore, it is important to be able to counsel these patients on the possibility of resuming high-demand activities, thus, we also included studies concerning these other osteotomy indications.

A limitation common to any systematic review is the risk of overlooking papers. However, we tried to overcome this with our extensive search strategy, which was conducted by an experienced clinical librarian (JD). Furthermore, we imposed no language restrictions and included French and Italian articles. A specific limitation to our systematic review is that the included studies showed a broad heterogeneity in terms of study design, study population, outcome measures, and overall quality. Thus, while this review presents the best available evidence on RTS and RTW after knee osteotomy, our results should be interpreted with caution. For example, preoperative or pre-symptomatic sports levels and work participation data were mostly collected postoperatively, which makes these findings prone to recall bias. Furthermore, many different secondary outcome measures for physical activity were used (e.g., Tegner score, Lysholm score, UCLA score), hampering comparisons of physical activity between studies. In addition, only a few studies corrected for confounding. For example, only 10 of 26 studies reported the mean BMI. This appears to be an important confounder since BMI >27.5 kg/m^2^ has been associated with worse outcomes, including worse activity levels, after knee osteotomies [66]. This implies that confounders that were not accounted for in the included studies may have influenced our findings. Future prospective studies should identify important confounders such as physical and mental comorbidities, preoperative sports levels and work status, patients’ motivation, and surgeon's advice, and should correct for these confounders in the analysis. Also, based on our extensive evaluation of the risk of bias, we found that studies with a low risk of bias reported lower percentages of RTS and RTW. This implies that future studies should carefully consider potential sources of bias and aim to account for these sources in the study design to find the most reliable percentages of RTS and RTW.

## Conclusion

The majority of patients undergoing knee osteotomy return to sports activities and work. For RTS, we observed a trend towards participation in lower-impact sports activities, similar to RTS after KA. Patients undergoing knee osteotomy returned to high-impact activities more often than did those undergoing KA.

For RTW, it appears that a return to the same or a higher workload is possible. This valuable information will aid both the orthopedic surgeon and the patient in the pre-operative decision-making process, and is especially interesting in the treatment of the younger, active, and employed OA population. The systematic comparison of current literature is hampered by the heterogeneity of patient populations, operative techniques, and an overall lack of accounting for possible confounding factors. Lastly, this review confirms the importance of using the pre-symptomatic level as a starting point when analyzing percentages of RTS and RTW.

## Electronic supplementary material

Below is the link to the electronic supplementary material.

Supplementary material 1 (XLSX 11 kb)

Supplementary material 2 (DOCX 16 kb)

Supplementary material 3 (DOCX 13 kb)
