# Supplementary material for: High Rates of Return to Sports Activities and Work After Osteotomies Around the Knee: A Systematic Review and Meta-Analysis
Source: Sports Med. 2017 Apr 11;47(11):2219–44. doi: 10.1007/s40279-017-0726-y (PMC5633634; doi:10.1007/s40279-017-0726-y)
Supplement: Supplementary file 2 — Supplementary material 2 (DOCX 16 kb) [file 40279_2017_726_MOESM2_ESM.docx]

## Electronic Supplementary Material Appendix S2. Criteria list for assessment of risk of bias.

1. **Study participation**

- Source population/population of interest is adequately described; low = fully mentioned, moderate = partially mentioned, high = not mentioned
- Clear description of power analysis; low = mentioned, high = not mentioned
- Recruitment period and place of recruitment of patients; low = fully mentioned, moderate = partially mentioned, high = not mentioned
- Description of inclusion and exclusion criteria; low = fully mentioned, moderate = partially mentioned, high = not mentioned
- Clear description of baseline characteristics: age, sex, body mass index, comorbidities and preoperative sports/work levels; low = fully mentioned, moderate = partially mentioned, high = not mentioned

1. **Study attrition, follow-up**

- Follow-up of at least 12 months; low = > 12 months, high = < 12 months
- Adequate response rate % loss to follow-up; low = < 20%, high = > 20%
- Information about non-responders versus responders: age, body mass index, comorbidities and preoperative sports level; low = fully mentioned, moderate = partially mentioned, high = not mentioned

1. **Prognostic factor measurement**

- A clear definition of performed osteotomy surgery; low =mentioned, high = not mentioned
- More specified description of type of osteotomy performed and any additional surgery (opening-wedge, closing-wedge, fixation method); low = fully mentioned, moderate = partially mentioned, high = not mentioned

1. **Outcome measurement**

- Clear definition of outcomes with special attention to definition of preoperative sports level, pre- and postoperative sports participation, return to sports (level and impact thereof), time to return to sports, satisfaction about activities and/or specific outcome measures for activity; low = mentioned, moderate = partly mentioned, high = not mentioned
- Valid and reliable measurement of outcomes are used, including blinding of outcome assessors; low = mentioned, moderate = partly mentioned high = not mentioned
- Same method and setting of outcome measurement for all study participants; low = fully mentioned and no large spreading of moments of outcome measurement; moderate = partially mentioned and/or large spreading of follow-up; high = not mentioned or different

1. **Study confounding**

- Important confounders mentioned: age, sex, BMI > 30, pre-operative sports/work level, restricting comorbidities and other restricting causes such as surgical complications, negative advice concerning RTS/RTW, precautions of psychological factors, rehabilitation; low = taken into account, moderate = partially mentioned, high = not taken into account at all
- Definition of the confounding factor; low = fully described, moderate = partially described, high = no description
- Valid and reliable measurement of confounders (e.g. may include relevant outside sources of information on measurement properties, also characteristics, such as blind measurement and limited reliance on recall); low = valid measurement of all confounders, moderate = valid measurement of some but not all confounders, high = invalid measurement or no confounding measurement
- Method and setting of confounding measurement; low = the same for all study participants, high = not the same for all study participants
- Appropriate accounting for confounding in study design and analysis; low = mentioned, moderate = partly mentioned, high = not mentioned

1. **Analysis and reporting**

- Clear presentation of analysis and significance of primary outcomes; low = fully mentioned, moderate = partially mentioned, high = not mentioned
- Authors reported use of one or more methods to reduce bias (standardisation, matching, adjustment in multivariate model, stratification, propensity scoring), assessed dose–response in some way (subgroup, regression) or justified sample size; low = fully mentioned, moderate = partially mentioned, high = not mentioned
- Reporting of all results, no selective reporting; low = no selective reporting, moderate = probably selective reporting, high = clearly selective reporting
