# Supplementary material for: High Rates of Return to Sports Activities and Work After Osteotomies Around the Knee: A Systematic Review and Meta-Analysis
Source: Sports Med. 2017 Apr 11;47(11):2219–44. doi: 10.1007/s40279-017-0726-y (PMC5633634; doi:10.1007/s40279-017-0726-y)
Supplement: Supplementary file 3 — Supplementary material 3 (DOCX 13 kb) [file 40279_2017_726_MOESM3_ESM.docx]

**Electronic Supplementary Material Appendix S3. Levels of impact on knee joint of different types of sports participation^a^**

| **Low-impact** | **Intermediate-impact** | **High-impact** |
| --- | --- | --- |
| Stationary cycling  Golf  Cross-country skiing  Swimming  Walking  Dancing  Water aerobics  Potentially Low:  Bowling  Fencing  Rowing  Isokinetic weight lifting  Sailing  Speed walking  Table tennis  Bicycling | Free weight lifting  Hiking  Horseback riding  Ice skating  Rock climbing  Low-impact aerobics  Doubles tennis  In-line skating  Downhill skiing | Baseball / softball  Basketball  Volleyball  Football  Handball  Racquetball  Jogging/ running  Lacrosse  Soccer  Singles tennis  Water skiing  Karate |

^a^According to Vail et al.
